# Influence of Demographic Factors on Clinical Outcomes in Adults With Chronic Idiopathic Constipation Treated With Plecanatide

**DOI:** 10.14309/ctg.0000000000000598

**Published:** 2023-05-10

**Authors:** Satish S.C. Rao, Adam P. Laitman, Philip B. Miner

**Affiliations:** 1 Division of Gastroenterology and Hepatology, Digestive Health Center, Augusta University, Augusta, Georgia, USA;; 2 Salix Pharmaceuticals, Bridgewater, New Jersey, USA;; 3 Oklahoma Foundation for Digestive Research (retired), Oklahoma City, Oklahoma, USA.

**Keywords:** bowel movement, constipation, demographics, guanylate cyclase-C agonist, plecanatide

## Abstract

Chronic idiopathic constipation (CIC) is a common condition that affects some patient groups more often. Demographic/clinical characteristics can differ in presentation and therapeutic response. The impact of these characteristics on plecanatide efficacy/safety was examined.

**METHODS:**

Data from 2 identically designed, randomized, phase 3 trials of adults with CIC receiving 3 mg of plecanatide, 6 mg of plecanatide, or placebo for 12 weeks were analyzed. Subgroups were baseline age, body mass index (BMI), race/ethnicity, and sex/gender. Endpoints included durable overall complete spontaneous bowel movement (CSBM) responder rate, weekly CSBMs and spontaneous bowel movements (SBMs), and adverse events.

**RESULTS:**

Overall (N = 2,639; 3 mg of plecanatide [n = 877]; 6 mg of plecanatide [n = 877]; and placebo [n = 885]), CSBM responder rates were significantly greater with 3 mg of plecanatide and 6 mg of plecanatide vs placebo in subgroups with those younger than 65 years (*P* < 0.001), females (*P* < 0.001), White individuals (*P* < 0.001), and BMI <25 kg/m^2^ (*P* ≤ 0.004) and 25–30 kg/m^2^ (*P* < 0.001); as well, for 3 mg: 65 years or older (*P* = 0.03), non-White individuals (*P* < 0.001), and BMI ≥30 kg/m^2^ (*P* = 0.02). Improvement from baseline in weekly CSBM and SBM frequency occurred in all subgroups for both plecanatide doses vs placebo (*P* ≤ 0.02) at week 12, except those aged 65 years or older for 6 mg of plecanatide. The most common adverse event was diarrhea (3 mg [4.9%]; 6 mg [5.4%]; and placebo [1.3%]).

**DISCUSSION:**

Pooled data from identically designed CIC trials strengthened the ability to identify meaningful subgroup comparisons regarding plecanatide efficacy and safety.

## Graphical abstract

**Figure:**
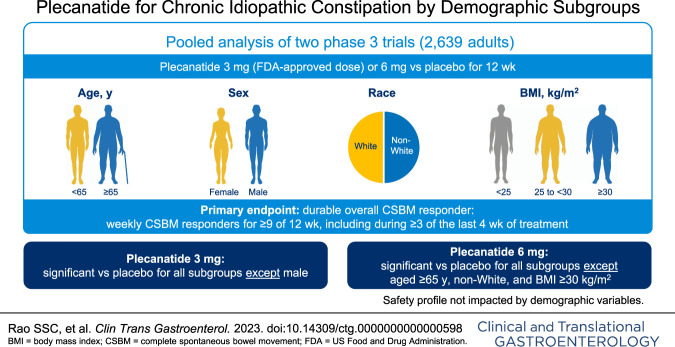

## INTRODUCTION

Chronic idiopathic constipation (CIC) is estimated to affect 5%–15% of the US population and up to 12% globally (1–3). It has been associated with decreased quality of life, disruptions in work productivity, and substantial healthcare costs (4–6). Demographic and clinical presentation differences influence the course and response to therapy of many disorders, including CIC (1,2). Several studies have reported differences in the prevalence of CIC among different demographic and ethnic subgroups in the community. One meta-analysis (n = 26 studies) reported a greater prevalence of CIC in female individuals compared with male individuals (17.4% vs 9.2%, respectively; odds ratio, 2.2; 95% confidence interval, 1.9–2.6) (1). A pooled population (United States, Canada, and the United Kingdom) data analysis indicated that constipation was more prevalent in younger individuals (aged 18–49 years) than older individuals (aged 50 years or older) (2); however, a systematic review (n = 16 of 68 relevant studies) concluded that the relationship between age and prevalence of constipation was variable (7). Findings from a retrospective, single-center study reported that female individuals and patients younger than 60 years with CIC experienced more severe symptoms than male individuals and older patients, suggesting that effects of treatment may vary depending on demographic and clinical symptom severity variables (8).

The influence of demographic variables on outcomes in chronic constipation has not been previously evaluated because of insufficient numbers of patients in each subgroup to ensure meaningful statistical analysis. After combining data from 2 identically designed trials, the number of patients in selected ethnic and demographic subgroups increased sufficiently to provide statistically meaningful treatment outcomes in patients with CIC (9,10). The demographic subgroup data for age, sex/gender, race/ethnicity, and body mass index (BMI) were pooled from 2 identically designed phase 3 trials of plecanatide for the treatment of CIC. Plecanatide is a pH-sensitive uroguanylin analog that acts locally as a guanylate cyclase-C agonist to increase electrolyte influx and net water secretion into the gastrointestinal lumen (11). Plecanatide 3 mg is indicated in the United States for the treatment of CIC and irritable bowel syndrome with constipation in adults (12). Previous randomized, placebo-controlled clinical trials have demonstrated that 3 and 6 mg of plecanatide were both efficacious and well tolerated in patients with CIC (13,14).

## METHODS

Data were pooled and analyzed post hoc from 2 identically designed, randomized, double-blind, placebo-controlled phase 3 trials in patients with CIC (ClinicalTrials.gov identifiers: NCT01982240 and NCT02122471). The study designs and patient populations, including the inclusion/exclusion criteria, have been previously described (13,14). In brief, the 2 trials included an eligibility screening period, a 12-week treatment period, and a 2-week follow-up period. Adults aged 80 years or younger with a BMI of 18–40 kg/m^2^ were eligible for inclusion if they met modified Rome III criteria (13,14) for functional constipation (CIC) for ≥3 months before screening, with symptom onset ≥6 months before diagnosis. Exclusion criteria included any structural abnormality of the gastrointestinal tract and conditions that could affect gastrointestinal motility or defecation, including a history or the presence of pelvic floor dysfunction or pseudo-obstruction. Patients were randomly assigned 1:1:1 (stratified by sex/gender) to receive 3 mg of oral plecanatide, 6 mg of oral plecanatide, or placebo, administered once daily for 12 weeks. Bowel movement information was documented daily by patients. Institutional review board approval was obtained for the 2 trials, and both were conducted in accordance with the International Conference on Harmonization Guidelines for Good Clinical Practice and the ethical principles of the Declaration of Helsinki.

Assessments included the protocol-specified primary and secondary endpoints from the 2 trials. A complete spontaneous bowel movement (CSBM) was defined as a spontaneous bowel movement (SBM) occurring in the absence of laxative use within 24 hours of bowel movement, with the patient reporting a sense of complete evacuation. A CSBM weekly responder was defined as a patient who had ≥3 CSBMs in a given week and an increase from baseline of ≥1 CSBM in the same week. The primary efficacy endpoint was the percentage of patients who were durable overall CSBM responders during 12 weeks of treatment, defined as patients who were weekly CSBM responders for ≥9 of the 12 weeks, including during ≥3 of the last 4 weeks of treatment. Efficacy endpoints were analyzed in patients subgrouped by age (younger than 65 years or aged 65 years or older), sex/gender (female or male), race/ethnicity (White or non-White individuals), or BMI (<25, 25 to <30, or ≥30 kg/m^2^). Adverse events (AEs) were monitored throughout the trials and evaluated by subgroup.

Efficacy analyses were conducted in the intention-to-treat population (defined as all nonduplicative patients who were randomly assigned to treatment). The safety analysis population included all patients in the intention-to-treat population who received at least 1 dose of study medication. For the primary efficacy endpoint, patients with missing data were considered nonresponders; plecanatide groups were compared with placebo using the Cochran-Mantel-Haenszel test, stratified by sex/gender; and 95% CIs were calculated using the Clopper-Pearson method. *P* values for changes from baseline in bowel movement frequency vs placebo were calculated from the pairwise comparison of least squares mean (LSM) values using an analysis of covariance model with fixed effects for sex/gender (stratification variable), treatment, week, and the interaction of treatment and week; in addition, a random intercept for patient for SBMs and fixed effects for treatment and covariates of sex/gender and corresponding baseline parameter values for SBMs. The model took into account the repeated measurements for each patient.

## RESULTS

**Figure 1.**
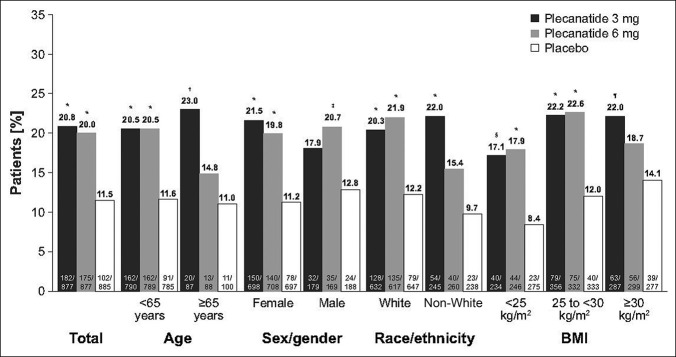
Percentage of patients with durable overall CSBM response for total population and by subgroup. *P ≤ 0.001 vs placebo. †*P* = 0.03 vs placebo. ‡*P* = 0.04 vs placebo. §*P* = 0.004 vs placebo. ¶*P* = 0.02 vs placebo. BMI, body mass index; CSBM, complete spontaneous bowel movement.

There were 2,639 patients with CIC in the combined trials (plecanatide 3 mg [n = 877], plecanatide 6 mg [n = 877], and placebo [n = 885]). The baseline demographic and clinical characteristics were comparable among the 3 patient groups (Table 1). Most of the 2,639 patients were younger than 65 years (89.6%), female (79.7%), White individuals (71.8%), and had a mean BMI of 28.2 kg/m^2^. In the overall population, the percentage of durable overall CSBM responders (primary efficacy endpoint) was significantly greater with 3 mg of plecanatide and 6 mg of plecanatide compared with placebo (*P* < 0.001 vs placebo for both; Figure 1), and this percentage was similar between the overall plecanatide 3 mg and 6 mg cohorts (approximately 20.0%).

**Table 1.** Demographic and baseline disease characteristics^a^

Characteristic	Plecanatide 3 mg (n = 877)	Plecanatide 6 mg (n = 877)	Placebo (n = 885)
Age, yr			
Mean (SD)	45.3 (14.6)	45.1 (14.1)	45.5 (14.3)
Range	18–80	18–80	18–80
Female, n (%)	698 (79.6)	708 (80.7)	697 (78.8)
Race/ethnicity, n (%)			
White	632 (72.1)	617 (70.4)	647 (73.1)
Black	211 (24.1)	206 (23.5)	194 (21.9)
Asian	20 (2.3)	29 (3.3)	27 (3.1)
Other	14 (1.6)	25 (2.9)	17 (1.9)
Mean BMI, kg/m^2^ (SD)	28.4 (5.0)	28.3 (5.1)	28.0 (5.2)
BMI subgroup, n (%)			
Underweight/normal (<25 kg/m^2^)	234 (26.7)	246 (28.1)	275 (31.1)
Overweight (25 to <30 kg/m^2^)	356 (40.6)	332 (37.9)	333 (37.6)
Obese (≥30 kg/m^2^)	287 (32.7)	299 (34.1)	277 (31.3)
Mean number of SBMs per wk (SD)	1.9 (1.9)	1.7 (1.7)	1.9 (1.8)
Mean number of CSBMs per wk (SD)	0.3 (0.6)	0.3 (0.5)	0.4 (0.5)

BMI, body mass index; CSBM, complete spontaneous bowel movement; SBM, spontaneous bowel movement.

a Intention-to-treat population.

When grouped by select baseline characteristics, a significantly greater percentage of patients treated with 3 mg of plecanatide were durable overall CSBM responders for most subgroups (Figure 1). These subgroups included those younger than 65 years or those aged 65 years or older (*P* ≤ 0.03), female individuals (*P* < 0.001), both race/ethnicity categories (*P* < 0.001), and all 3 BMI classifications (*P* ≤ 0.02); a statistically significant difference was not observed for male individuals (Figure 1). Statistically significant differences favoring 6 mg of plecanatide vs placebo were observed for all subgroups except ages 65 years or older, non-White individuals, and BMI ≥30 kg/m^2^ (Figure 1).

**Figure 2.**
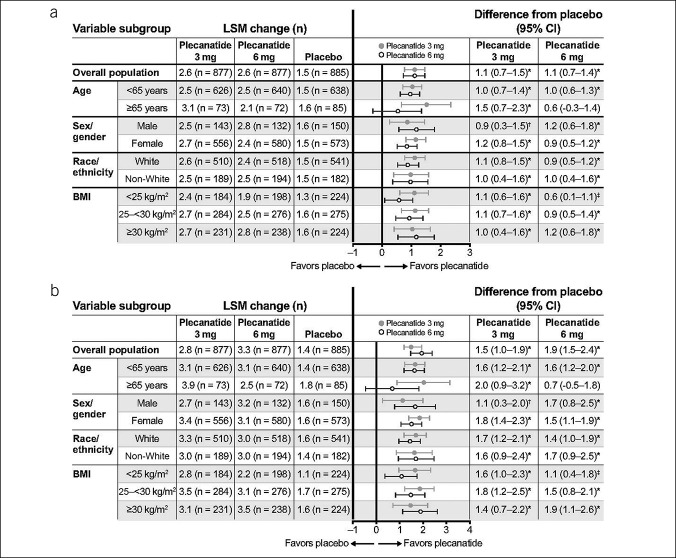
Change from baseline in (**a**) weekly CSBMs and (**b**) weekly SBMs in overall population and subgroups at week 12. *P ≤ 0.001 vs placebo. †*P* ≤ 0.01 vs placebo. ‡*P* = 0.02 vs placebo. BMI, body mass index; CSBM, complete spontaneous bowel movement; LSM, least squares mean; SBM, spontaneous bowel movement.

In the overall population, the LSM change from baseline in the weekly number of CSBMs at week 12 was significantly improved with 3 mg of plecanatide (Δ 2.6) and 6 mg of plecanatide (Δ 2.6) vs placebo (Δ 1.5; *P* < 0.001 vs placebo for both plecanatide doses; Figure 2). The weekly LSM number of SBMs was significantly improved from baseline to week 12 with 3 mg of plecanatide (Δ 2.8) and 6 mg of plecanatide (Δ 3.3) compared with placebo (Δ 1.4; *P* < 0.001 vs placebo for both plecanatide doses). In addition, in the overall population, significant improvement from baseline with plecanatide treatment for both weekly CSBM and SBM frequency was observed as early as week 1 and continued weekly through week 12. For each of the subgroup analyses (by age, sex, race/ethnicity, and BMI), significant improvement from baseline in CSBM frequency with 3 mg of plecanatide and 6 mg of plecanatide vs placebo was observed as early as week 1 (see Supplementary Digital Content, http://links.lww.com/CTG/A941, which shows LSM change from baseline for each subgroup by week). In addition, statistically significantly greater LSM changes from baseline in weekly CSBMs and SBMs were observed at week 12 with 3 mg of plecanatide in all subgroups (*P* ≤ 0.02; Figure 2). For 6 mg of plecanatide, significant differences were also observed for all subgroups at week 12 except patients aged 65 years or older (*P* ≤ 0.02; Figure 2).

### Safety

The safety analysis population included 2,627 patients (3 mg of plecanatide [n = 875], 6 mg of plecanatide [n = 877], and placebo [n = 875]). The subgroup analyses revealed no new safety issues with plecanatide in any specific subgroup, and the AE profiles were not affected by demographic variables (Tables 2–4). In the overall population, the most common AEs reported with 3 mg of plecanatide and 6 mg of plecanatide vs placebo were diarrhea (4.9% and 5.4%, vs 1.3%), headache (1.8% and 1.8%, vs 2.1%), and nasopharyngitis (1.0% and 2.3%, vs 1.6%). In the subgroup analyses, diarrhea was reported in <5.0% of patients treated with 3 mg of plecanatide, except for those aged 65 years or older (5.7%), female individuals (5.9%), White individuals (5.2%), and those with BMI <25 kg/m^2^ (5.1%). Rates of diarrhea in the plecanatide 6 mg treatment group were reported in ≥5.0% of those younger than 65 years (5.2%), those aged 65 years or older (6.7%), female individuals (5.8%), White individuals (5.0%), non-White individuals (6.2%), and those with a BMI ≥30 kg/m^2^ (7.0%).

**Table 2.** Adverse event profile in patients with CIC subgrouped by age

Patients, n (%)	Plecanatide 3 mg		Plecanatide 6 mg		Placebo	
	<65 yr (n = 788)	≥65 yr (n = 87)	<65 yr (n = 788)	≥65 yr (n = 89)	<65 yr (n = 777)	≥65 yr (n = 98)
Any AE	240 (30.5)	34 (39.1)	250 (31.7)	33 (37.1)	231 (29.7)	26 (26.5)
Discontinuation due to an AE	31 (3.9)	6 (6.9)	36 (4.6)	5 (5.6)	15 (1.9)	5 (5.1)
Discontinuation due to diarrhea	19 (2.4)	2 (2.3)	22 (2.8)	2 (2.2)	2 (0.3)	2 (2.0)
Most common AEs^a^						
Diarrhea	38 (4.8)	5 (5.7)	41 (5.2)	6 (6.7)	9 (1.2)	2 (2.0)
Headache	14 (1.8)	2 (2.3)	13 (1.6)	3 (3.4)	16 (2.1)	2 (2.0)
UTI	12 (1.5)	2 (2.3)	12 (1.5)	1 (1.1)	14 (1.8)	2 (2.0)
URTI	9 (1.1)	3 (3.4)	3 (0.4)	2 (2.2)	10 (1.3)	0
Nausea	8 (1.0)	0	10 (1.3)	0	12 (1.5)	1 (1.0)
Abdominal distension	8 (1.0)	2 (2.3)	7 (0.9)	1 (1.1)	2 (0.3)	1 (1.0)
Flatulence	7 (0.9)	2 (2.3)	6 (0.8)	2 (2.2)	6 (0.8)	0
Nasopharyngitis	9 (1.1)	0	18 (2.3)	2 (2.2)	14 (1.8)	0
Cough	8 (1.0)	0	3 (0.4)	2 (2.2)	4 (0.5)	0
Influenza	6 (0.8)	0	8 (1.0)	1 (1.1)	7 (0.9)	0
Arthralgia	5 (0.6)	2 (2.3)	3 (0.4)	1 (1.1)	3 (0.4)	1 (1.0)
Abdominal pain	5 (0.6)	1 (1.1)	11 (1.4)	0	7 (0.9)	1 (1.0)
Vomiting	5 (0.6)	1 (1.1)	4 (0.5)	0	2 (0.3)	2 (2.0)
Upper abdominal pain	5 (0.6)	0	3 (0.4)	0	2 (0.3)	2 (2.0)
Hypertension	5 (0.6)	0	2 (0.3)	1 (1.1)	5 (0.6)	2 (2.0)
Extremity pain	1 (0.1)	1 (1.1)	1 (0.1)	0	0	2 (2.0)
Fall	1 (0.1)	0	1 (0.1)	0	1 (0.1)	2 (2.0)

AE, adverse event; CIC, chronic idiopathic constipation; URTI, upper respiratory tract infection; UTI, urinary tract infection.

a AEs, occurring in ≥1.0% of patients in any subgroup and with any treatment. Ordered by percentage frequency in overall plecanatide 3 mg group and then alphabetical.

**Table 3.** Adverse event profile in patients with CIC subgrouped by race/ethnicity or sex/gender

Patients, n (%)	Race/ethnicity					
	Plecanatide 3 mg		Plecanatide 6 mg		Placebo	
	White (n = 633)	Non-White (n = 242)	White (n = 617)	Non-White (n = 260)	White (n = 638)	Non-White (n = 237)
Any AE	204 (32.2)	70 (28.9)	198 (32.1)	85 (32.7)	177 (27.7)	80 (33.8)
Discontinuation due to an AE	25 (3.9)	12 (5.0)	26 (4.2)	15 (5.8)	15 (2.4)	5 (2.1)
Discontinuation due to diarrhea	15 (2.4)	6 (2.5)	15 (2.4)	9 (3.5)	3 (0.5)	1 (0.4)
Most common AEs^a^						
Diarrhea	33 (5.2)	10 (4.1)	31 (5.0)	16 (6.2)	9 (1.4)	2 (0.8)
Headache	10 (1.6)	6 (2.5)	12 (1.9)	4 (1.5)	16 (2.5)	2 (0.8)
URTI	8 (1.3)	4 (1.7)	4 (0.6)	1 (0.4)	4 (0.6)	6 (2.5)
Nasopharyngitis	8 (1.3)	1 (0.4)	10 (1.6)	10 (3.8)	7 (1.1)	7 (3.0)
Abdominal distension	5 (0.8)	5 (2.1)	3 (0.5)	5 (1.9)	2 (0.3)	1 (0.4)
Abdominal pain	3 (0.5)	3 (1.2)	7 (1.1)	4 (1.5)	6 (0.9)	2 (0.8)
Anemia	1 (0.2)	0	3 (0.5)	2 (0.8)	1 (0.2)	3 (1.3)

Patients, n (%)	Sex/gender					
	Plecanatide 3 mg		Plecanatide 6 mg		Placebo	
	Female (n = 695)	Male (n = 180)	Female (n = 707)	Male (n = 170)	Female (n = 691)	Male (n = 184)
Any AE	226 (32.5)	48 (26.7)	239 (33.8)	44 (25.9)	220 (31.8)	37 (20.1)
Discontinuation due to an AE	33 (4.7)	4 (2.2)	34 (4.8)	7 (4.1)	18 (2.6)	2 (1.1)
Discontinuation due to diarrhea	20 (2.9)	1 (0.6)	21 (3.0)	3 (1.8)	4 (0.6)	0
Most common AEs^a^						
Diarrhea	41 (5.9)	2 (1.1)	41 (5.8)	6 (3.5)	11 (1.6)	0
UTI	14 (2.0)	0	13 (1.8)	0	15 (2.2)	1 (0.5)
Headache	13 (1.9)	3 (1.7)	13 (1.8)	3 (1.8)	17 (2.5)	1 (0.5)
Nasopharyngitis	7 (1.0)	2 (1.1)	18 (2.5)	2 (1.2)	12 (1.7)	2 (1.1)

AE, adverse event; CIC, chronic idiopathic constipation; URTI, upper respiratory tract infection; UTI, urinary tract infection.

a AEs, occurring in ≥1.0% of patients in any subgroup and with any treatment. Ordered by percentage frequency in overall plecanatide 3 mg group and then alphabetical.

**Table 4.** Adverse event profile in patients with CIC subgrouped by BMI

n (%)	Plecanatide 3 mg			Plecanatide 6 mg			Placebo		
	<25 kg/m^2^ (n = 234)	25 to <30 kg/m^2^ (n = 356)	≥30 kg/m^2^ (n = 285)	<25 kg/m^2^ (n = 244)	25 to <30 kg/m^2^ (n = 334)	≥30 kg/m^2^ (n = 299)	<25 kg/m^2^ (n = 273)	25 to <30 kg/m^2^ (n = 328)	≥30 kg/m^2^ (n = 274)
Any AE	73 (31.2)	103 (28.9)	98 (34.4)	76 (31.1)	100 (29.9)	107 (35.8)	83 (30.4)	87 (26.5)	87 (31.8)
Discontinuation due to an AE	12 (5.1)	15 (4.2)	10 (3.5)	8 (3.3)	16 (4.8)	17 (5.7)	3 (1.1)	9 (2.7)	8 (2.9)
Due to diarrhea	7 (3.0)	7 (2.0)	7 (2.5)	5 (2.0)	8 (2.4)	11 (3.7)	1 (0.4)	1 (0.3)	2 (0.7)
Most common AEs^a^									
Diarrhea	12 (5.1)	17 (4.8)	14 (4.9)	11 (4.5)	15 (4.5)	21 (7.0)	6 (2.2)	2 (0.6)	3 (1.1)
Headache	5 (2.1)	4 (1.1)	7 (2.5)	4 (1.6)	5 (1.5)	7 (2.3)	3 (1.1)	11 (3.4)	4 (1.5)
UTI	5 (2.1)	6 (1.7)	3 (1.1)	4 (1.6)	3 (0.9)	6 (2.0)	6 (2.2)	5 (1.5)	5 (1.8)
URTI	4 (1.7)	3 (0.8)	5 (1.8)	2 (0.8)	2 (0.6)	1 (0.3)	1 (0.4)	5 (1.5)	4 (1.5)
Sinusitis	2 (0.9)	3 (0.8)	7 (2.5)	1 (0.4)	3 (0.9)	2 (0.7)	0	2 (0.6)	1 (0.4)
Abdominal distension	5 (2.1)	0	5 (1.8)	2 (0.8)	2 (0.6)	4 (1.3)	3 (1.1)	0	0
Nasopharyngitis	6 (2.6)	3 (0.8)	0	9 (3.7)	6 (1.8)	5 (1.7)	8 (2.9)	4 (1.2)	2 (0.7)
Flatulence	3 (1.3)	3 (0.8)	3 (1.1)	4 (1.6)	1 (0.3)	3 (1.0)	2 (0.7)	3 (0.9)	1 (0.4)
Nausea	3 (1.3)	1 (0.3)	4 (1.4)	1 (0.4)	4 (1.2)	5 (1.7)	5 (1.8)	1 (0.3)	7 (2.6)
Vomiting	3 (1.3)	0	3 (1.1)	2 (0.8)	0	2 (0.7)	1 (0.4)	1 (0.3)	2 (0.7)
Abdominal pain	2 (0.9)	2 (0.6)	2 (0.7)	1 (0.4)	5 (1.5)	5 (1.7)	5 (1.8)	2 (0.6)	1 (0.4)
Back pain	0	2 (0.6)	3 (1.1)	2 (0.8)	3 (0.9)	3 (1.0)	5 (1.8)	1 (0.3)	6 (2.2)

AE, adverse event; BMI, body mass index; CIC, chronic idiopathic constipation; URTI, upper respiratory tract infection; UTI, urinary tract infection.

a AEs, occurring in ≥1.0% of patients in any subgroup and with any treatment. Ordered by percentage frequency in overall plecanatide 3 mg group and then alphabetical.

## DISCUSSION

CIC has been reported to affect some demographic groups more than others, such as female individuals and the older population more often than male or younger individuals (1,2,7). Despite recognition of the influence of demographic subgroups on the prevalence of constipation, a paucity of information exists regarding the clinical and therapeutic outcomes in these subgroups. As healthcare moves toward precision medicine and personalized treatment (15,16), this information can be helpful to tailor treatment approaches to the appropriate population. The demographic subgroups of age, sex/gender, race/ethnicity, and BMI were evaluated for plecanatide safety and efficacy in a post hoc analysis of 2 large phase 3 CIC trials (13,14).

In patients with CIC subgrouped by age (younger than 65 years and aged 65 years or older), sex/gender (female and male individuals), race/ethnicity (White and non-White individuals), and BMI subcategorized into 3 groups (<25 kg/m^2^, 25 to <30 kg/m^2^, and ≥30 kg/m^2^), those treated with 3 mg of plecanatide and 6 mg of plecanatide had significantly higher durable overall CSBM response rates compared with placebo for most statistical comparisons. These analyses demonstrated concordance for efficacy and safety endpoints for each subgroup when compared with the overall response observed with the pooled data. In addition, the change from baseline to week 12 in the weekly number of CSBMs or SBMs was significantly greater with 3 mg of plecanatide and 6 mg of plecanatide compared with placebo for patients younger than 65 years, female individuals, male individuals, White and non-White patients, and all 3 BMI categories that were examined. The subgroup of patients aged 65 years or older had a significantly greater change from baseline to week 12 in CSBMs/wk and SBMs/wk with 3 mg of plecanatide compared with placebo, but not with 6 mg of plecanatide. This partly may be related to the smaller number of patients included in this age subgroup (<100 per group). In addition, there may have been an imbalance in the number of patients who had taken constipating agents or had an undiagnosed evacuation disorder. No new or subgroup-specific AEs were identified in the current post hoc analysis. Overall, plecanatide as a treatment option for CIC was safe and efficacious across subgroups and showed results comparable to those reported separately for each clinical trial (13,14).

Limitations of this analysis include those inherent to the nature of post hoc and exploratory analyses. Combining the 2 trials was justified because of parallel study designs, geographic participation, treatment dosing and duration, and patient baseline characteristics. Despite the general increase in subgroup sample size by pooling of data, some subgroups were limited in size, which may have precluded the ability to detect differences between plecanatide and placebo. In addition, we analyzed a limited number of demographic variables, and future studies should evaluate additional factors as well.

In conclusion, although CIC is associated with differences in the prevalence of demographic and clinical characteristics, such as age, sex/gender, race/ethnicity, and BMI, these pooled analyses showed that the plecanatide clinical efficacy profile was superior to placebo, and the safety profile of plecanatide was comparable across all subgroups.

## CONFLICTS OF INTEREST

**Guarantor of the article:** Satish S.C. Rao, MD, PhD.

**Specific author contributions:** S.S.C.R., A.P.L., and P.B.M.: participated in the research of the article, had access to the data, and participated in the preparation of the article. All authors read and approved the final version of the manuscript.

**Financial support:** The trials were supported by Synergy Pharmaceuticals, and the post hoc analyses were supported by Salix Pharmaceuticals.

**Potential competing interests:** S.S.C.R. has served on advisory boards for Medtronic, Takeda Pharmaceuticals, and Salix Pharmaceuticals. A.P.L. is an employee of Salix Pharmaceuticals. P.B.M. reports having nothing to disclose.

**IRB approval statement:** Approvals were obtained for 1 trial (NCT01982240) from a centralized IRB (Schulman Associates IRB) and 4 site IRBs (Chesapeake IRB; NorthShore University HealthSystem IRB; University of Oklahoma Health Sciences Center IRB; and Western IRB). Approvals were obtained for 1 trial (NCT02122471) from a centralized IRB (Copernicus Group) and 1 site IRB (University of Oklahoma Health Sciences Center IRB).

**Study Highlights**

**WHAT IS KNOWN**

✓ Demographic variables affect the prevalence of chronic constipation in the community.
✓ Whether treatment of chronic constipation is influenced by ethnic, demographic, and clinical factors is unknown.
✓ Published studies are inadequately powered for meaningful subgroup analyses.

**WHAT IS NEW HERE**

✓ Subgroup analyses of combined data from 2 identical clinical trials of plecanatide were performed.
✓ Plecanatide was safe and efficacious across several variables, including age, sex/gender, race/ethnicity, and body mass index.
✓ Plecanatide efficacy profile was superior to placebo, and the plecanatide safety profile was comparable across subgroups.
✓ Subgroup analysis enriches the understanding of the pharmacophysiology of plecanatide.
✓ This analysis provides a template for designing prospective analyses of subgroups for identically designed registration trials.

## Supplementary Material
