# Supplementary material for: Influence of Demographic Factors on Clinical Outcomes in Adults With Chronic Idiopathic Constipation Treated With Plecanatide
Source: Clin Transl Gastroenterol. 2023 May 10;14(7):e00598. doi: 10.14309/ctg.0000000000000598 (PMC10371318; doi:10.14309/ctg.0000000000000598)
Supplement: Supplementary file 1 [file ct9-14-e00598-s001.pdf]

Supplemental Digital Content

Supplemental Figure 1. Change from baseline in weekly CSBMs in patients <65 years of age.

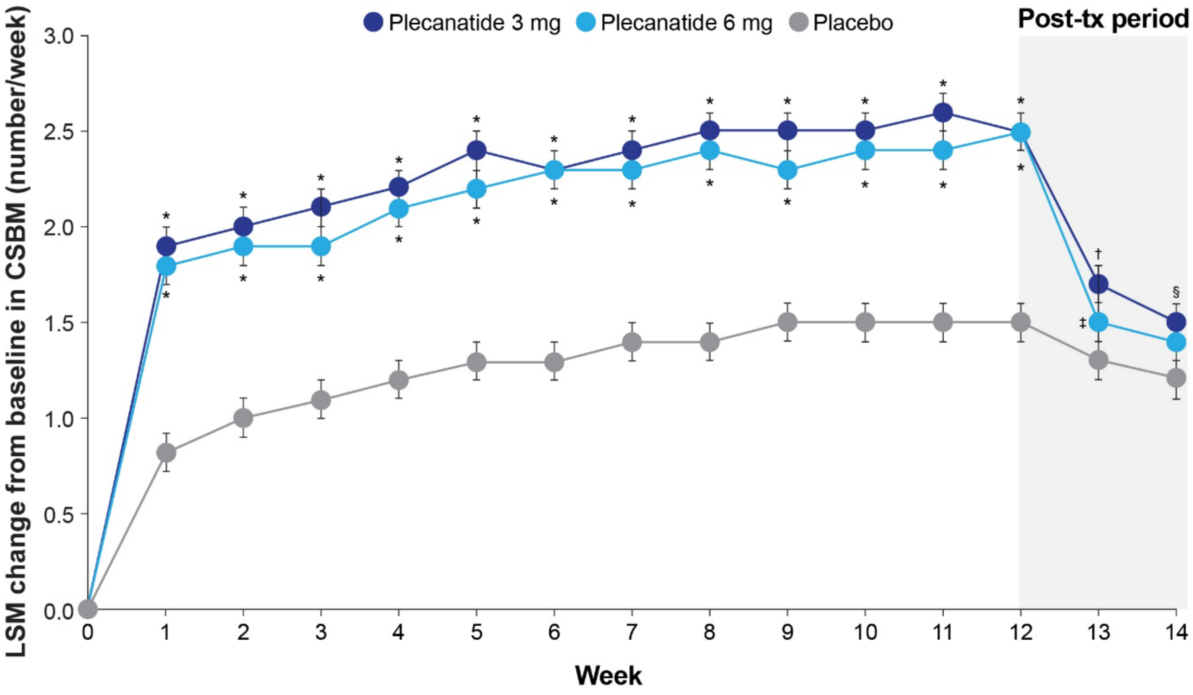

CSBM, complete spontaneous bowel movement; LSM, least squares mean.

*$P < 0.001$ vs. placebo. †$P < 0.01$ vs. placebo. ‡$P = 0.046$ vs. placebo. §$P = 0.02$ vs. placebo.

Supplemental Figure 2. Change from baseline in weekly CSBMs in patients ≥65 years of age.

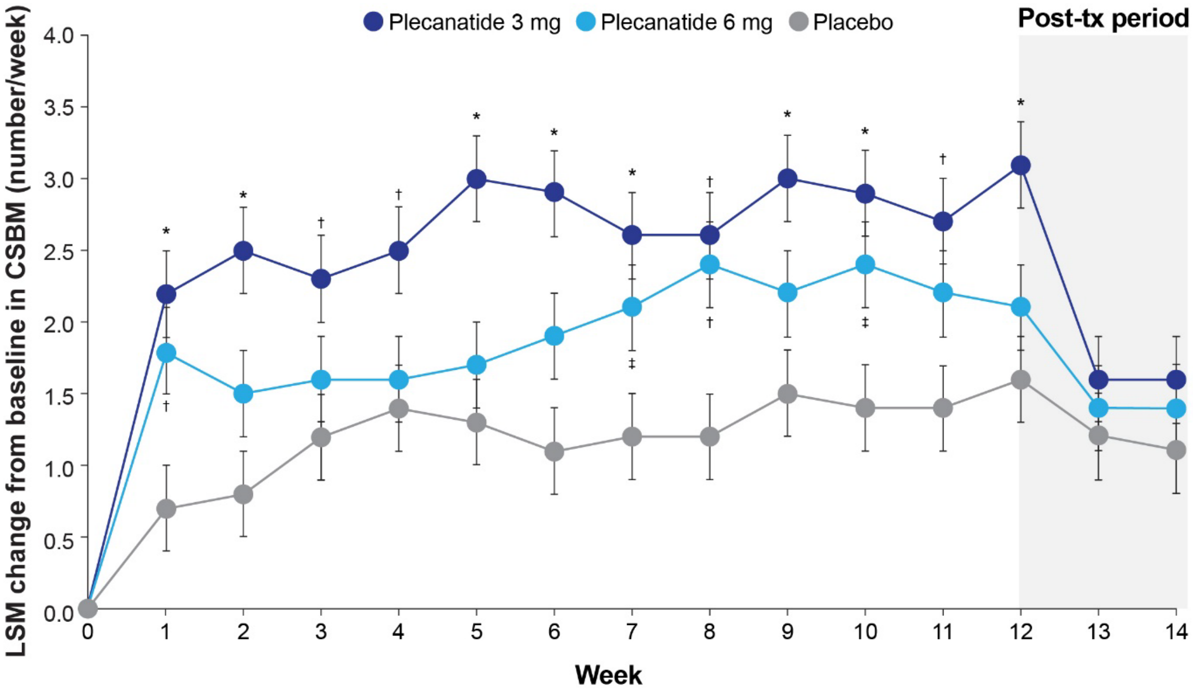

CSBM, complete spontaneous bowel movement; LSM, least squares mean.

*$P < 0.001$ vs. placebo. †$P < 0.01$ vs. placebo. ‡$P = 0.02$ vs. placebo.

Supplemental Figure 3. Change from baseline in weekly CSBMs in males.

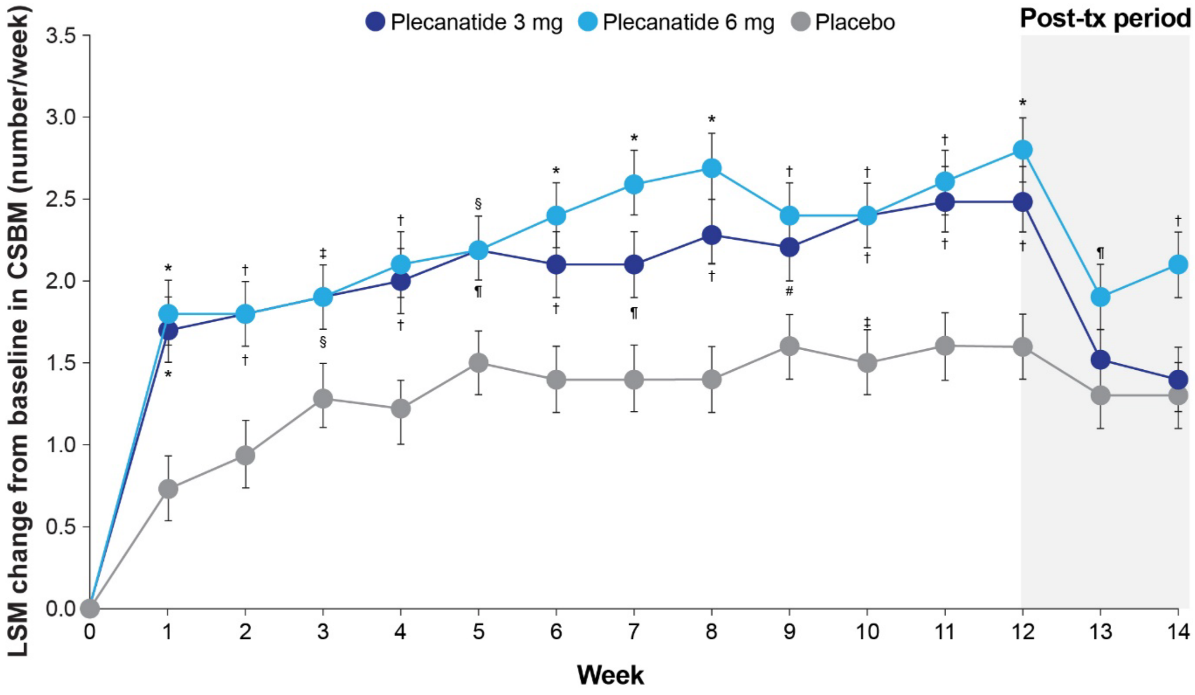

CSBM, complete spontaneous bowel movement; LSM, least squares mean.

*$P \leq 0.001$ vs. placebo. †$P \leq 0.01$ vs. placebo. ‡$P = 0.046$ vs. placebo. §$P = 0.02$ vs. placebo. ¶$P = 0.03$ vs. placebo. #$P = 0.04$ vs. placebo.

Supplemental Figure 4. Change from baseline in weekly CSBMs in females.

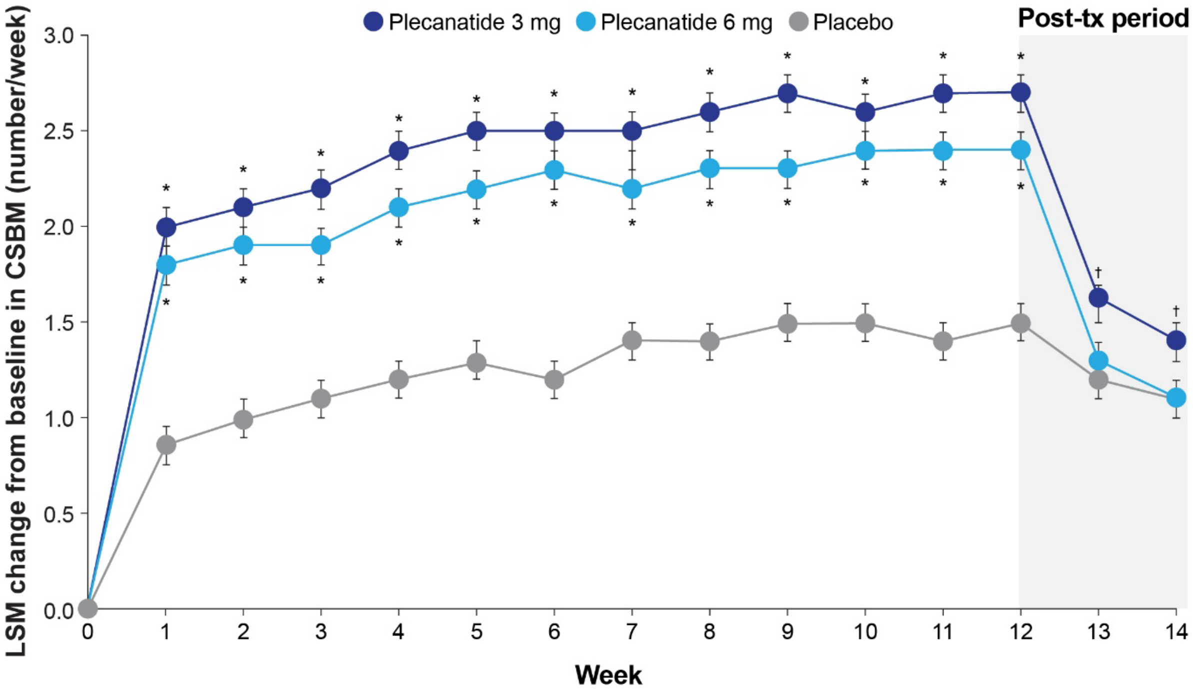

CSBM, complete spontaneous bowel movement; LSM, least squares mean.

*$P < 0.001$ vs. placebo. †$P < 0.01$ vs. placebo.

Supplemental Figure 5. Change from baseline in weekly CSBMs for white race/ethnicity.

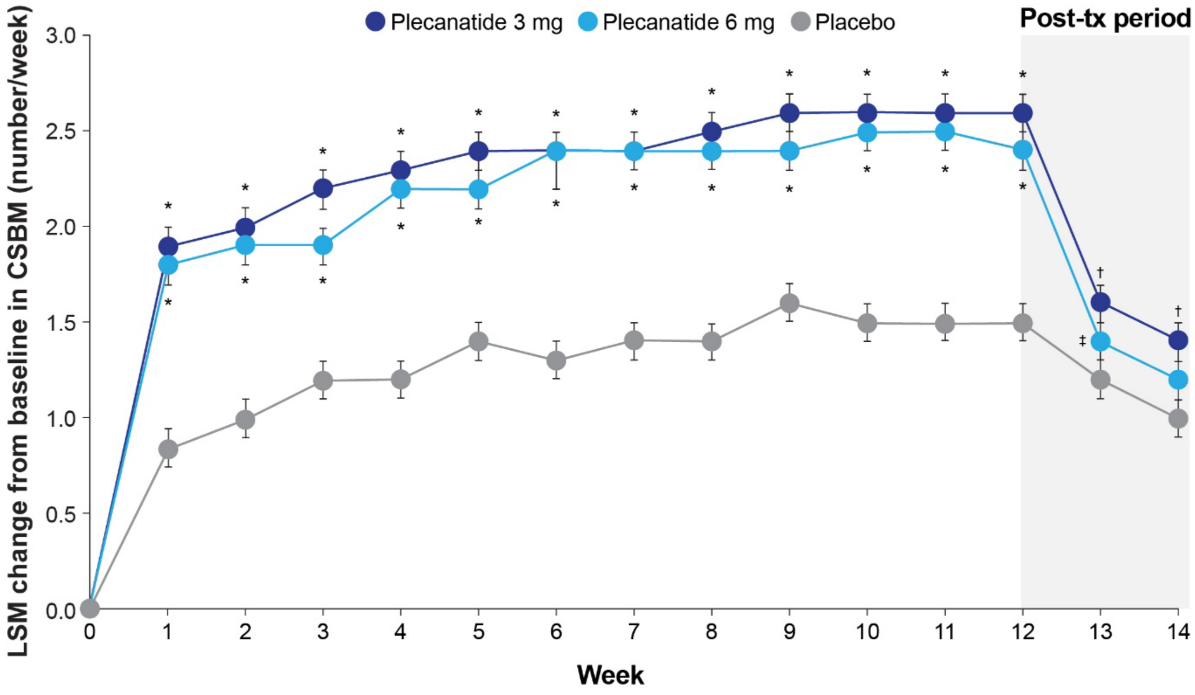

CSBM, complete spontaneous bowel movement; LSM, least squares mean.

[*]$P < 0.001$ vs. placebo. [†]$P \leq 0.01$ vs. placebo. [‡]$P = 0.046$ vs. placebo.

Supplemental Figure 6. Change from baseline in weekly CSBMs for non-white race/ethnicity.

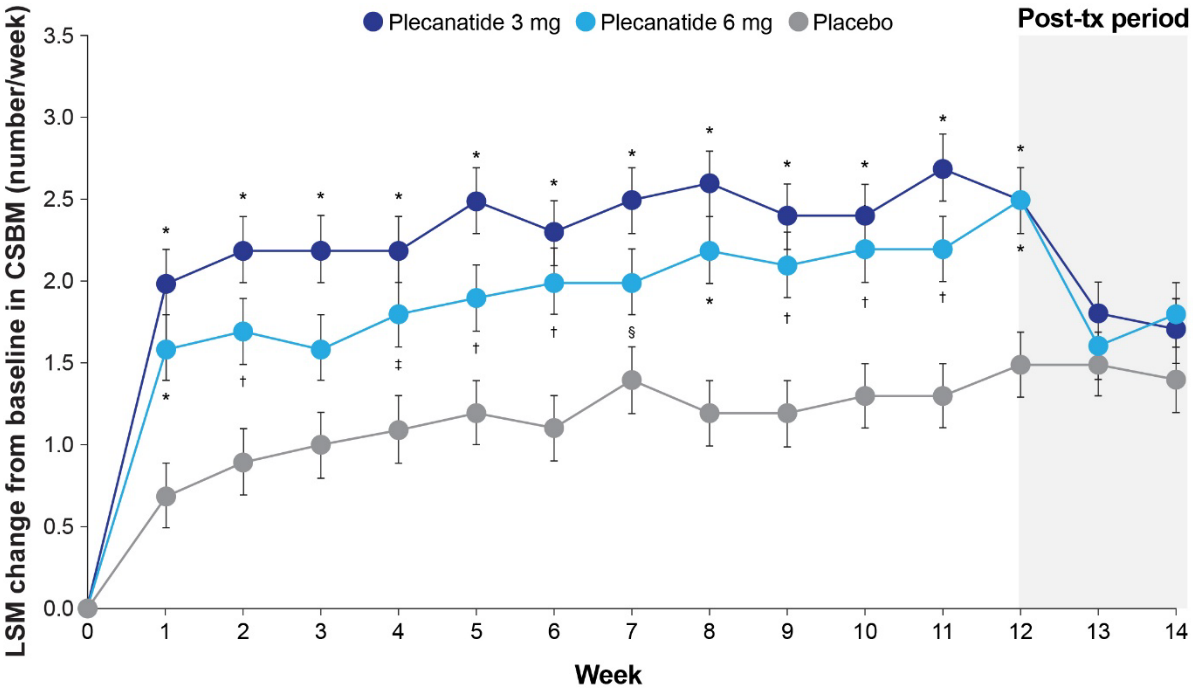

CSBM, complete spontaneous bowel movement; LSM, least squares mean.

*$P \leq 0.001$ vs. placebo. †$P \leq 0.01$ vs. placebo. ‡$P = 0.02$ vs. placebo. §$P = 0.04$ vs. placebo.

Supplemental Figure 7. Change from baseline in weekly CSBMs for patients with a BMI <25 kg/m.

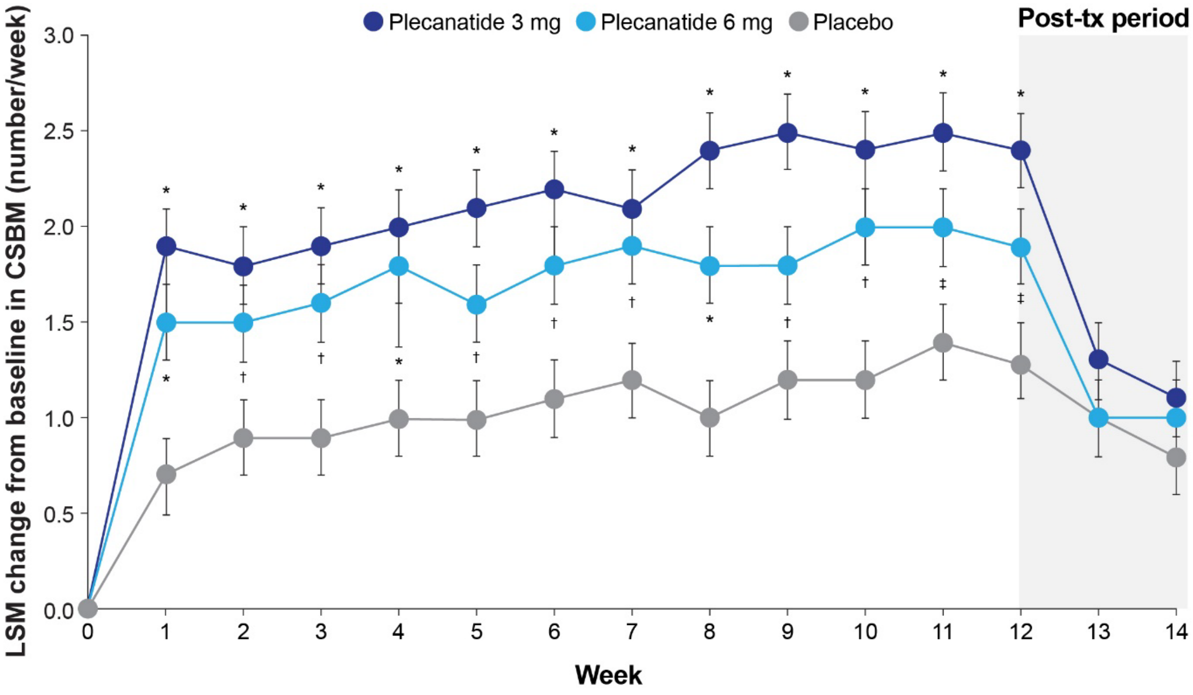

BMI, body mass index; CSBM, complete spontaneous bowel movement; LSM, least squares mean.

*$P \leq 0.001$ vs. placebo. †$P \leq 0.01$ vs. placebo. ‡$P = 0.02$ vs. placebo.

Supplemental Figure 8. Change from baseline in weekly CSBMs for patients with a BMI 25 to <30 kg/m.

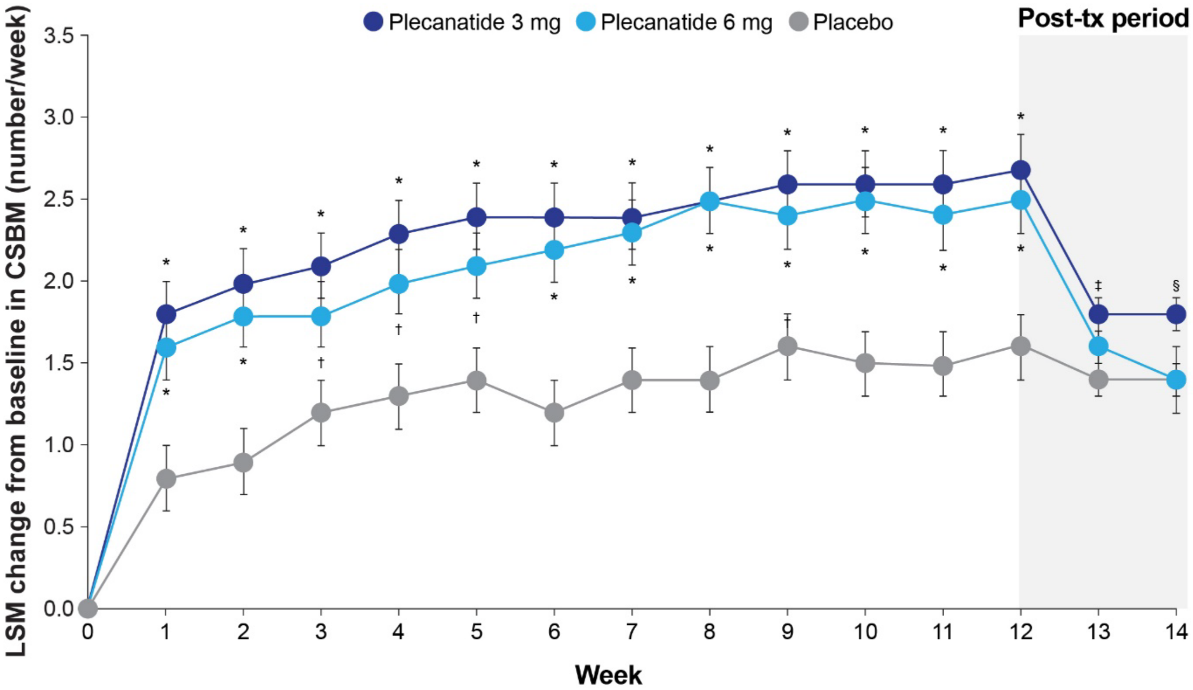

BMI, body mass index; CSBM, complete spontaneous bowel movement; LSM, least squares mean.

*$P < 0.001$ vs. placebo. †$P \leq 0.01$ vs. placebo. ‡$P = 0.02$ vs. placebo. §$P = 0.04$ vs. placebo.

Supplemental Figure 9. Change from baseline in weekly CSBMs for patients with a BMI ≥30 kg/m^2.

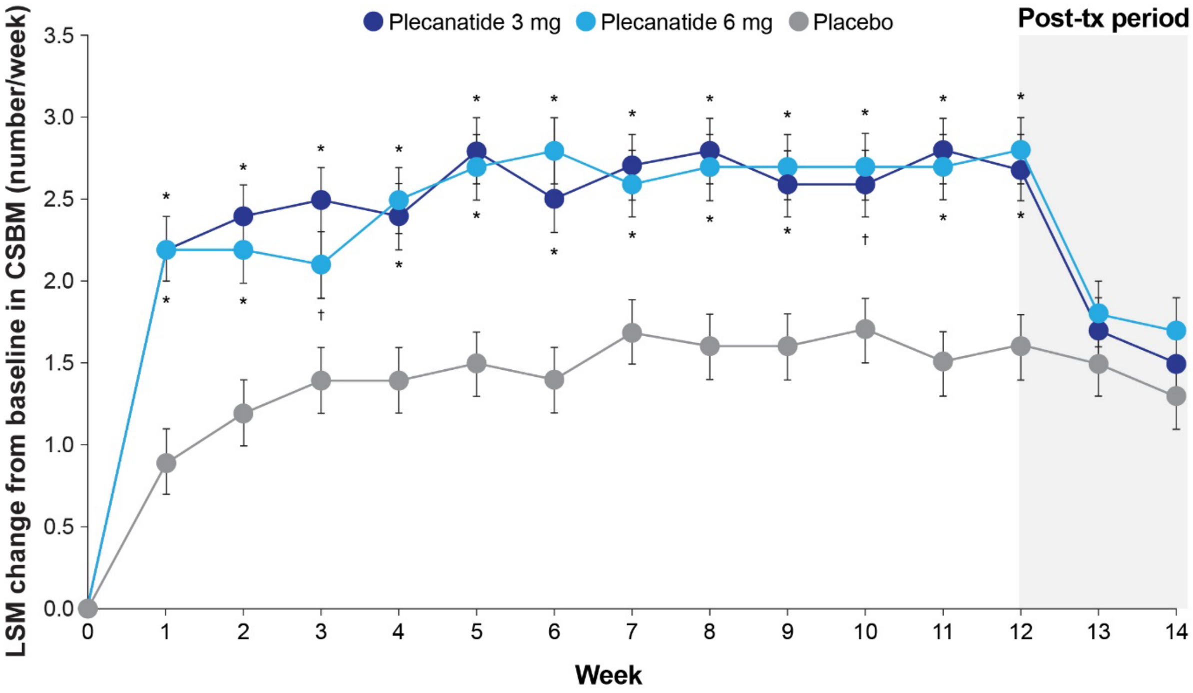

BMI, body mass index; CSBM, complete spontaneous bowel movement; LSM, least squares mean.

[*]$P \leq 0.001$ vs. placebo. [†]$P \leq 0.01$ vs. placebo.
